# Thyrotropin serum levels and coexistence with Hashimoto’s thyroiditis as predictors of malignancy in children with thyroid nodules

**DOI:** 10.1186/s13052-019-0693-z

**Published:** 2019-08-06

**Authors:** Giuseppina Zirilli, Giuseppina Salzano, Domenico Corica, Giovanni Battista Pajno, Cristina Mignosa, Giorgia Pepe, Filippo De Luca, Giuseppe Crisafulli

**Affiliations:** Department of Human Pathology of Adulthood and Childhood Unit of Pediatrics, University of Messina, Messina, Italy

**Keywords:** Childhood, Nodular lymphocytic thyroiditis, Pre-cancerous factors, Subclinical hypothyroidism, TSH serum levels

## Abstract

Thyroid cancer (TC) in childhood is a rare disease characterized by an excellent prognosis. Thyroid nodules in children, although less common than in adults, have a greater risk of malignancies, particularly in those cases associated with anamnestic, clinical and ultrasonographic risk factors.

Among the factors, which have been found to be linked with an increased relative risk of TC in children, an important role seems to be possibly played by an underlying nodular Hashimoto’s thyroiditis (HT) and by the serum levels of TSH.

Aim of this Commentary was to specifically address this last point.

According to the available pediatric literature on the relationships between these risk factors and phenotypical expression of TC in children, it is possible to conclude that: 1) It is not completely clarified if HT per se predisposes to malignancy or if it represents an incidental histologic finding in cases with TC or if it may be the result of an immune response against tumoral cells. 2) It is unclear whether phenotypic expression of TC is more severe in the cases with associated HT but normal TSH serum levels. 3) Persistently elevated TSH levels play an independent role as predictors of the likelihood of TC, especially in children but also in adults. 4) Patients with nodular HT and subclinical hypothyroidism need to be treated with Levothyroxine in order to prevent the development of both TC and severe thyroid dysfunctions.

## Introduction

Thyroid cancer (TC) in childhood is a rare disease characterized by an excellent prognosis. Thyroid nodules are known to be less common in children (estimated prevalence 0.2–5%) than in adults (estimated prevalence 19–35%) [1]. Nevertheless, a greater risk of malignancies of pediatric thyroid nodules compared to those in adults (22–26% versus 5–10%) has been reported, particularly in those cases associated with clinical and ultrasonographic (US) risk factors [1–4]. Epithelial-derived differentiated thyroid cancer (DTC), including papillary (PTC) and follicular thyroid cancer (FTC), occurs at more advanced stages in children than in adults and it is associated with a higher rates of recurrence, early lymph node involvement and metastases (most commonly lung metastases) [3]. However, even in presence of metastatic disease, a 30-yr survival rates of 90–99% for children with DTC has been reported, likely since the major part of cases are well-differentiated tumor and good-responder to adjuvant radioactive iodine (RAI) therapy [3].

Family history of thyroid carcinoma, genetic syndrome (i.e. Cowden’s syndrome, Gardner syndrome, Werner syndrome), mutations in the RET protooncogene, previous neck irradiation, and the presence of specific nodule features as growing and very firm nodule, fixation of the nodule to adjacent structures, hoarseness and cervical lymphadenopathy, have been considered risk factors for malignant evolution of thyroid nodules in pediatrics [2, 3]. Furthermore, ultrasound appearance of solid lesions, characterized by hypoechogenecity, microcalcifications, irregular margins, no halo, intranodular vascularity, and lesions taller than wide, have been reported as potential predictors of thyroid malignancy [2, 5].

Genetics of TC differs between children and adults. While in adults RAS, BRAF, PAX8 or PPARG genes mutations are frequently identified in PTC, these mutations are very rare in pediatric PTCs in which prevail RET/PTC, AKAP9–BRAF, and NRK1 rearrangements [6]. The most frequently observed rearrangements in pediatric PTC are RET/PTC gene ones, particularly in the variants RET/PTC1 and RET/PTC3, determining a constitutive activation of several pathways, as the mitogen-activated protein kinase (MAP kinase), phospholipase C (PLC) and phospho inositide 3 kinase–protein kinase B (PI3K–Akt) cascades, able to stimulate thyroid follicular cell proliferation and survival, and to inhibit cell differentiation [6]. Genetic differences between pediatric and adult PTCs, could explain, at least partially, the better response to RAI therapy in children with PTC, their low mortality and rare progression to less-differentiated tumors [1].

Due to these relevant peculiarities, pediatric TC has been suggested by many authors to be considered as a distinct clinical entity, which should deserve specific diagnostic and therapeutic recommendations [3, 4, 7].

However, data concerning the predictors of malignancy in pediatric population are limited by both the infrequency of thyroid nodules in this age and the small cohorts described so far. Furthermore, available data are usually based on retrospective cross-sectional studies and, frequently, on surgical series that are affected by a selection bias due to the high risk of malignancy of patients who underwent surgery.

According to the interesting review by Mussa et al. [8], aiming to specifically evaluate the diagnostic accuracy of various features of children’ thyroid nodules in assessing the likelihood of malignancy, it seems that none of the examined clinical, laboratory and US parameters has a diagnostic accuracy as high as that of fine-needle aspiration biopsy (FNAB). However, that study evidenced that male sex, compression symptoms, specific lymph node alterations, microcalcifications, indistinct nodule margins and hypoechoic pattern, with increased intranodular vascularization at ultrasound, were significantly associated with malignancy [8]. By contrast, regular margins, mixed echoic pattern and peripheral-only vascularization were associated with benignity [8]. Therefore, it may be argued, from those data, that clinical, laboratory and US features of nodules could be employed in pediatric age as reliable predictors of either malignancy or benignity, thus representing a preliminary step to identify the children to submit to FNAB.

Other factors, which have been found to be significantly linked with an increased risk of TC in children, are both the coexistence with Hashimoto’s thyroiditis HT [9, 10] and having a persistent increase in TSH serum levels [11].

Aim of the present Commentary is to specifically address this last point, i.e. the role played by these two risk factors in conditioning a malignant outcome in children and adolescents with thyroid nodules.

### Coexistence of thyroid nodules with HT as a predictor of TC

HT is an autoimmune organ-specific disease characterized by a wide spectrum of morphological gland alterations, which may have different repercussions on thyroid function. In fact, at the time of HT presentation, hormonal patterns may range from euthyroidism to subclinical or overt hypothyroidism and even transient hyperthyroidism, at least in pediatric age [12–17].

Its pathogenesis is based on an abnormal immune response against thyroid autoantigens, with the contribution of genetic and environmental factors [18].

The relative prevalence of TC among patients with HT is a matter of controversy, both in adults and in children. Available data reported a wide variability of prevalence of association between HT and TC (up to 85%) [19], depending above all on cohort features (i.e. ages, gender distribution, surgical series).

According to a meta-analysis on the specific issue of correlations between HT and TC, the occurrence rate of histologically confirmed HT in adult patients with PTC seems to be 2.8 times higher than that detected in individuals with benign thyroid disorders and 2.4 times higher than in patients with other types of TC [19]. According to the same meta-analysis, including 2471 adult cases with histologically proven HT from 38 eligible studies, PTCs with coexisting HT were significantly related to female gender, multifocal involvement, no extra-thyroidal extension and no lymph node metastasis. Furthermore, the coexistence of HT in PTCs was significantly linked with a long recurrence-free survival [19]. Based on the results of this meta-analysis, these authors suggested a close relationship between HT and PTC and recommended a careful monitoring for HT patients. However, it was not completely clarified if a pre-existing lymphocytic infiltration due to HT could facilitate the development of PTC or, conversely, lymphocytic infiltration of HT is the results of an immune reaction to tumor-specific antigens from a pre-existing PTC [19].

**Fig. 1:**
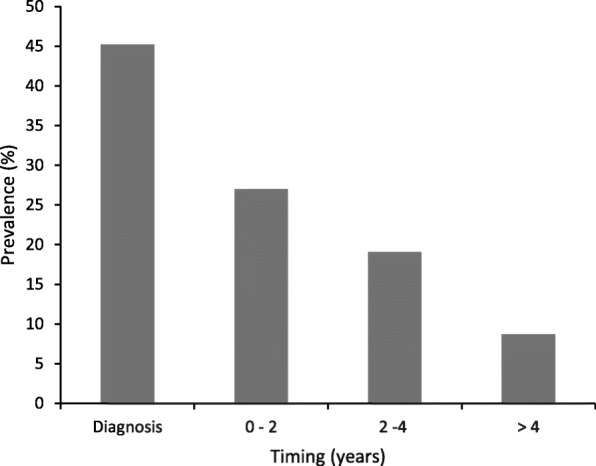
Timing of nodule discovery relative to diagnosis of Hashimoto’s thyroiditis (HT) in a cohort of 115 children with nodular HT (according to the findings of reference [20])

According to the wide variability of TC prevalence in HT, in a cohort of HT children and adolescents, a 3% of prevalence of TC has been reported [20]. In this pediatric series, the findings of lymphadenopathy and progressive increase in nodule diameter under Levothyroxine (L-T4) therapy are two factors which seem to be significantly associated with the risk of malignancy [20]. In most children thyroid nodules may be detected already at the time of HT diagnosis but it is not infrequent that they are discovered later (Fig. 1). In other pediatric multicenter cohort, involving children and adolescents affected by HT, a very low prevalence of TC (1.1%) compared to other series has been reported [21]; these author did not demonstrate influence of TSH levels on TC occurrence, likely due to the small number of patients with TC in their cohort [21]. Furthermore, Kambalapalli et al. [22] reported a 4% of prevalence of TC in a group of children and adolescents with goiter without statistical difference between positive and negative thyroid peroxidase antibody (TPOAb) patients.

A predisposing role of concomitant HT has also been taken into consideration to explain the more aggressive TC presentation in children and adolescents than in young adults aged between 20 and 30 years, recently reported [23]; indeed, in that study an association between TC and HT was found to be more common in children. Furthermore, in this study an increased prevalence of biochemical thyroid function alterations, compatible with overt hypothyroidism, has been demonstrated in the group of children, thereby these authors hypothesized that HT could exert a promoting effect on TC growth, at least partially, through a secondary chronic elevation of TSH serum levels [23], as previously reported [18, 24, 25]. The complex interrelations between thyroid autoimmunity and cancer phenotypical expression have been investigated also by other authors and it was postulated that HT might exert a promoting effect on TC growth, either directly or through a secondary TSH elevation [18, 24, 25].

Recent views on this issue, therefore, support the idea that both adults and children with HT may be potentially more prone to the risk of developing a differentiated TC with invasive features [9, 10, 24, 25]. This risk seems to increase with increasing TSH and thyroid autoantibody serum levels [25].

Whatever the causative relationship between thyroid autoimmunity and gland malignancy, HT may favor an early detection of TC, which can be diagnosed at a less progressed stage in the cases with pre-existing HT [9]. Likely, this is the main reason why patients with the association HT-TC may often exhibit a better outcome than those without HT [19], although TC in these individuals is potentially more aggressive and invasive, at least at the time of clinical presentation [10, 23].

In conclusion, it is sufficiently accepted that there is a relationship between HT and PTC, but it remains unclear whether HT predisposes to malignancy or it represents an incidental histologic finding in cases with TC or, on the contrary, whether it may be the result of a systemic/localized immune system response against tumoral cells [26]. A hypothetical causal link between HT and PTC is the creation of a favorable setting for malignant deterioration [27]. By contrast, it has also been postulated that the autoimmune response may counteract cancer expansion [26]. Finally, it has to be considered that a previous diagnosis of HT can stimulate an increased attention to thyroid gland, thus facilitating an earlier diagnosis of cancer. This can concur to explain the apparent paradox that patients with HT are more exposed to the risk of TC but exhibit also favorable clinic-pathologic features of their malignancy, when compared to those with TC but no HT.

### Increased TSH serum levels as predictors of TC

The association between HT and TC may also be mediated by a persistent increase in TSH serum levels, in the context of the biochemical picture of either subclinical or overt hypothyroidism, which is often observed at HT presentation [13, 14] and might be responsible for an overstimulation of follicular cell proliferation and growth. Several studies, in fact, support a significant role of TSH as risk factor for DTC in both children and adults [11, 24, 25, 28].

According to the study by Kim et al. [28], including 2131 euthyroid adult patients with PTC who underwent surgery, pre-operative TSH serum levels, although included in the normal range, were significantly higher in the study group than in age-matched control subjects drawn from the general population [28]. These authors concluded that having a high TSH level, even if included in normal range, may be considered as an independent predictor of DTC, regardless of age, gender and family history [28]. Furthermore, they inferred that TSH values in individuals with thyroid nodules may be used as diagnostic adjuncts for the selection of high-risk patients, who may need further investigations and/or surgery [28]. Accordingly, a large meta-analysis reported a positive association between TC diagnosis and serum TSH level [29]. Particularly, the model elaborated by these authors predicts a three times greater odds of TC being present in a patient with a serum TSH level of 4 mU/liter compared with one with a serum TSH of 0 mU/liter, a doubling of the odds of TC in relation to a serum TSH variation within normal range (between 0.65 and 4 mU/liter), and a doubling of the odds of TC in case of TSH increase from normal to elevated serum levels (from 2.2 to 7 mU/liter) [29]. Nevertheless, the cross-section design of meta-analyzed studies should be considered a limitation of these results in evaluating the causative role of TSH in TC pathogenesis, as suggested by these authors [29].

At this point a well-founded question is whether autoimmunity per se, or rather high TSH levels as consequence of HT are responsible for the increased frequency of differentiated TC in the patients with HT and thyroid nodules [24]. According to the results of the pediatric study by Mussa et al. [8], including children with isolated thyroid nodules and no associated HT, the risk of having a malignant nodule increased progressively from the first to the fifth quintile of TSH serum levels and, particularly, exhibited a 16-fold increase in those children with a TSH level above the median value for that series compared to others. Furthermore, mean TSH levels were significantly higher in children with malignant nodules than in those with benign nodules [8]. Based on these results, it was possible to argue that TSH levels per se play an autonomous role as predictors of the likelihood of TC in pediatric age [8].

Although an increased risk of TC in children and adolescents with nodular HT is still debated and not constantly reported [8, 21], it seems likely that increased levels of TSH, a consequence of the thyroid damage by the autoimmune process, could be a factor of risk of TC.

Therefore, L-T4 treatment in patients with nodular HT may have a role in slowing the progression of nodular lesions and the development of clinically detectable TC [24].

This last inference is especially appropriate for children with both nodular HT and a concomitant biochemical picture of subclinical hypothyroidism [30]. In fact, children with HT-related subclinical hypothyroidism are known to be more inclined to develop, over time, a severe thyroid dysfunction [16, 17]. Furthermore, in these cases also the odds of developing a TC are enhanced by the coexistence between HT and persistently elevated TSH serum levels. Therefore, whereas L-T4 therapy is not indicated in asymptomatic children with mild and idiopathic subclinical hypothyroidism, it is, on the contrary, highly advisable in children with HT-related subclinical hypothyroidism and persistent TSH elevation [30].

## Conclusions

1. It is not completely clarified if HT per se predisposes to malignancy or if it represents an incidental histologic finding in cases with TC or if it may be the result of an immune response against tumoral cells.
2. It is unclear whether phenotypic expression of TC is more severe in cases with associated HT but normal TSH serum levels.
3. Persistently elevated TSH levels play an independent role as predictors of the likelihood of TC, especially in children but also in adults.
4. Patients with nodular HT and subclinical hypothyroidism need to be treated with L-T4 in order to prevent the development of both TC and severe thyroid dysfunctions.

## Data Availability

Data sharing not applicable to this article as no datasets were generated or analyzed during the current study.

## Abbreviations

FNAB: Fine-needle aspiration biopsy
HT: Hashimoto’s thyroiditis
TC: Thyroid cancer
US: Ultrasonographic
